# Transcriptome and metabolome profiling provide insights into molecular mechanism of pseudostem elongation in banana

**DOI:** 10.1186/s12870-021-02899-6

**Published:** 2021-03-01

**Authors:** Guiming Deng, Fangcheng Bi, Jing Liu, Weidi He, Chunyu Li, Tao Dong, Qiaosong Yang, Huijun Gao, Tongxin Dou, Xiaohong Zhong, Miao Peng, Ganjun Yi, Chunhua Hu, Ou Sheng

**Affiliations:** 1 Institute of Fruit Tree Research, Guangdong Academy of Agricultural Sciences, Guangzhou, China; 2 Key Laboratory of South Subtropical Fruit Biology and Genetic Resource Utilization, Ministry of Agriculture, Guangzhou, China; 3 Key Laboratory of Tropical and Subtropical Fruit Tree Research, Guangdong Province, Guangzhou, China; 4 Horticulture and Landscape College, Hunan Agricultural University, Changsha, 410128 China

**Keywords:** Auxin efflux carrier proteins, Banana Pseudostem, Dwarfism, Ethylene response factors, Gibberellins

## Abstract

**Background:**

Banana plant height is an important trait for horticultural practices and semi-dwarf cultivars show better resistance to damages by wind and rain. However, the molecular mechanisms controlling the pseudostem height remain poorly understood. Herein, we studied the molecular changes in the pseudostem of a semi-dwarf banana mutant Aifen No. 1 (*Musa* spp. Pisang Awak sub-group ABB) as compared to its wild-type dwarf cultivar using a combined transcriptome and metabolome approach.

**Results:**

A total of 127 differentially expressed genes and 48 differentially accumulated metabolites were detected between the mutant and its wild type. Metabolites belonging to amino acid and its derivatives, flavonoids, lignans, coumarins, organic acids, and phenolic acids were up-regulated in the mutant. The transcriptome analysis showed the differential regulation of genes related to the gibberellin pathway, auxin transport, cell elongation, and cell wall modification. Based on the regulation of gibberellin and associated pathway-related genes, we discussed the involvement of gibberellins in pseudostem elongation in the mutant banana. Genes and metabolites associated with cell wall were explored and their involvement in cell extension is discussed.

**Conclusions:**

The results suggest that gibberellins and associated pathways are possibly developing the observed semi-dwarf pseudostem phenotype together with cell elongation and cell wall modification. The findings increase the understanding of the mechanisms underlying banana stem height and provide new clues for further dissection of specific gene functions.

**Supplementary Information:**

The online version contains supplementary material available at 10.1186/s12870-021-02899-6.

## Background

In banana (*Musa* spp.) production, plant architecture offers unique horticultural practices including, flower removal, bunch management, controlling pests and diseases, etc. High banana pseudostem is easily broken in a typhoon and needs additional input cost for propping. Productivity optimization could be achieved by manipulating banana plant height. Semi-dwarf banana cultivars show good resistance to damages caused by heavy wind and rain [1]. Furthermore, the yield gain associated with shorter stems is also linked to an increased harvest index [2]. In spite of the basic and strategic importance of semi-dwarf stem, the progress on genetic manipulation of banana plant height has been comparatively limited, partially because the ontogenesis of the pseudostem is not as easily characterized as for other organs, and its external morphological complexity is not so attractive as compared to leaves, roots, and flowers [3].

In vitro techniques are often applied in the banana industry for producing large-scale disease-free plantlets with higher yield potential. However, as a result of such processing, somaclonal variations often appear, which are considered as one of the major disadvantages for such techniques [4]. Dwarfism and giant variants are often observed in banana tissue culture plantlets [1, 5, 6]. On the other hand, the variants derived from tissue culture processing, however, are a good source of research materials suitable for a comparative transcriptome and metabolome analysis. Pisang Awak (*Musa* spp.) is a cultivated banana sub-group with high yield and superior taste quality, which is widely grown in Asian countries as a desert fruit but for juice/brewing in African countries [7]. Normally, the plants of Pisang Awak varieties are vigorous with a height around 4.5–5.5 m. In our *Musa* collections, there is a dwarf Pisang Awak variety ‘Aifen No. 1’ (wild type, WT), of which the height is around 2.0–2.5 m. Recently, a semi-dwarf mutant (MT) with the height 2.8–3.5 m, was identified by screening the somaclonal variations from ‘Aifen No. 1’ tissue cultured plants. Previous studies showed that hormone biosynthesis pathways are responsible for such variations [1].

So far, the key pathways that have been associated with the stem height are the gibberellic acid (GA) metabolism and signaling, elasticity of the cell-wall of expanding cells, and cross-talk between GA and other hormone metabolism and signaling pathways i.e. auxin, brassinosteroids (BR), and abscisic acid (ABA) [3]. Chen et al., [1] studied a dwarf mutant of ‘Williams’ banana variety and demonstrated the role of GA in dwarfism. In peach trees, the brachytic dwarfism trait (*dw*) was associated with a nonsense mutation in a GA receptor *PpeGID1c* [8]. GA biosynthesis pathway-related enzymes such as *ent*-Copalyl diphosphate synthase, *ent*-Kaurene synthase, *ent*-Kaurene 19-oxidase, GA12-aldehyde 7-oxidase, GA 20-oxidase, GA 3β-hydroxylase, and GA 2-oxidase have been successfully identified in Arabidopsis, pumpkin, and runner bean [9]. A previous study on GA metabolisms between Williams banana and its dwarf mutant shed light on the GA regulated stem development in banana and identified six main genes regulating differential GA content [1]. While this study focused on GA metabolism-related genes, the other pathways related to stem elongation remain poorly explored [9, 10].

In plants, the stem is initiated at shoot apical meristem (SAM) and in the case of seed plants, it is originated from the sub-apical region (called rib zone, RZ) of SAM [11]. Additionally, the monocot stem elongation is promoted by intercalary meristems (IM). Both RZ and IMs are the main sites where stem growth starts under GA stimuli [3]. GA degrades DELLA proteins and stimulates the cell division in RZ zone. DELLAs, the proteins controlling cell division in the stem, are functionally associated with transcription factors (TFs) e.g. DELLAs bind and inhibit the activity of class 1 teosinte branched 1 (TCP), cycloidea, and proliferating cell factor TFs, which further activates other genes associated with cell cycle progression in stem [12]. Recent studies have highlighted the direct link of DELLAs and cell prolification in RZ, which not only influences stem elongation but also the SAM size. Other than DELLAs, organ boundary genes such as lateral organ boundaries (LOB) domain-containing genes, have also been implicated in stem elongation by restricting signals that control stem elongation [13]. The establishment of vascular pattern in the early stages of stem development is a key stage and is under the influence of auxin flow. In this regard, the role of auxin efflux carrier proteins has been associated with the cell elongation in root epidermal cells but such a role in stem elongation has not been explored yet [14]. After the initial growth based on cell prolification, cell expansion comes into action for internode growth. Stem growth is adjusted to changeable conditions by the rapid differential growth. For cell prolification as well as expansion, the prominent role of cell-wall related genes is also an important consideration. Finally, the coordination between tissue layers through signaling also plays an important role during stem elongation. The role of auxin in BR production and synthesis as well as the signaling between BR and very long-chain fatty acids in the epidermis are good examples [15, 16].

With the availability of the banana genome, it is now possible to gain a deeper insight into the mechanisms governing plant height [17–19]. Unbiased modern high-throughput technologies such as whole transcriptome and metabolome have geared up the discovery of genetic factors controlling different traits in plants including, biotic and abiotic stresses, and agronomic traits [20–23]. Furthermore, the combination of multiple omics techniques has resulted in the elucidation of different pathways in banana such as peel ripening and the effect of melatonin on delaying anthracnose incidence [24–26]. In this study, we aimed at identifying the differentially expressed genes and metabolites in pseudostems of Pisang Awak Aifen No. 1 dwarf wild type and its semi-dwarf mutant. The transcriptome and metabolome responses involved hormonal signaling including GA, auxin, BR, ABA, and ethylene, and cell prolification and elongation.

## Results

### Plant phenotype

**Fig. 1:**
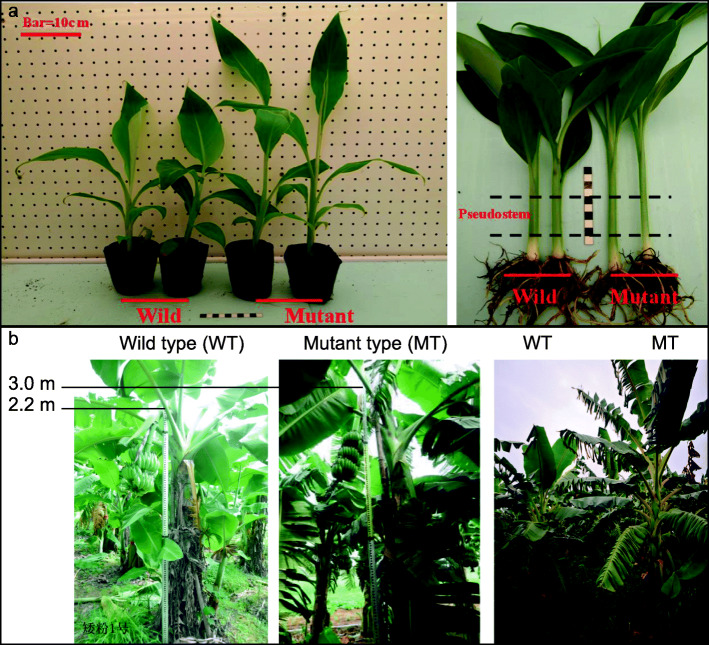
**a**) Six-week old wild type and semi-dwarf mutant plants of Aifen No. 1. The second panel shows the pseudostems used for combined omics analysis and **b**) comparison of plant height

In the field, the adult dwarf wild type (WT) banana Aifen No.1 (Musa spp. ABB group) plant presented short pseudostems ranging from 2.0–2.5 m with the stem girth ranging from 95.5–98.5 cm. In addition, the bunch weight of WT was 15–20 kg. The identified Aifen No.1 mutant (MT) plant had a semi-dwarf stature with 2.8–3.5 m plant height, 92.5–95.0 cm stem girth, and a relatively heavier bunch i.e. 25–30 kg (Table 1; Fig. 1a&b).

**Table 1:** Variation in stem height and stem girth at different time points of studied WT and MT Aifen No. banana plants

Time	Trait	Wild type (Dwarf)	Mutant (High)
**5–6 leaves seedlings**	Stem girth (cm)	3.5–5.0	2.0–3.0
	Plant height (m)	0.12–0.14	0.18–0.22
**Three months**	Stem girth (cm)	20.3–22.5	15.5–18.5
	Plant height (m)	0.4–0.8	1.0–1.3
**Six months**	Stem girth (cm)	45.5–49.5	38.5–40.6
	Plant height (m)	1.0–1.2	1.5–2.0
**Nine months**	Stem girth (cm)	80.6–82.5	74.5–76.8
	Plant height (m)	1.6–1.8	2.5–3.0
**Budding stage**	Stem girth (cm)	95.5–98.5	92.5–95.0
	Plant height (m)	2.0–2.5	2.8–3.5
**Harvest period**	Stem girth (cm)	95.5–98.5	92.5–95.0
	Plant height (m)	2.0–2.5	2.8–3.5

### Transcriptome analysis

**Fig. 2:**
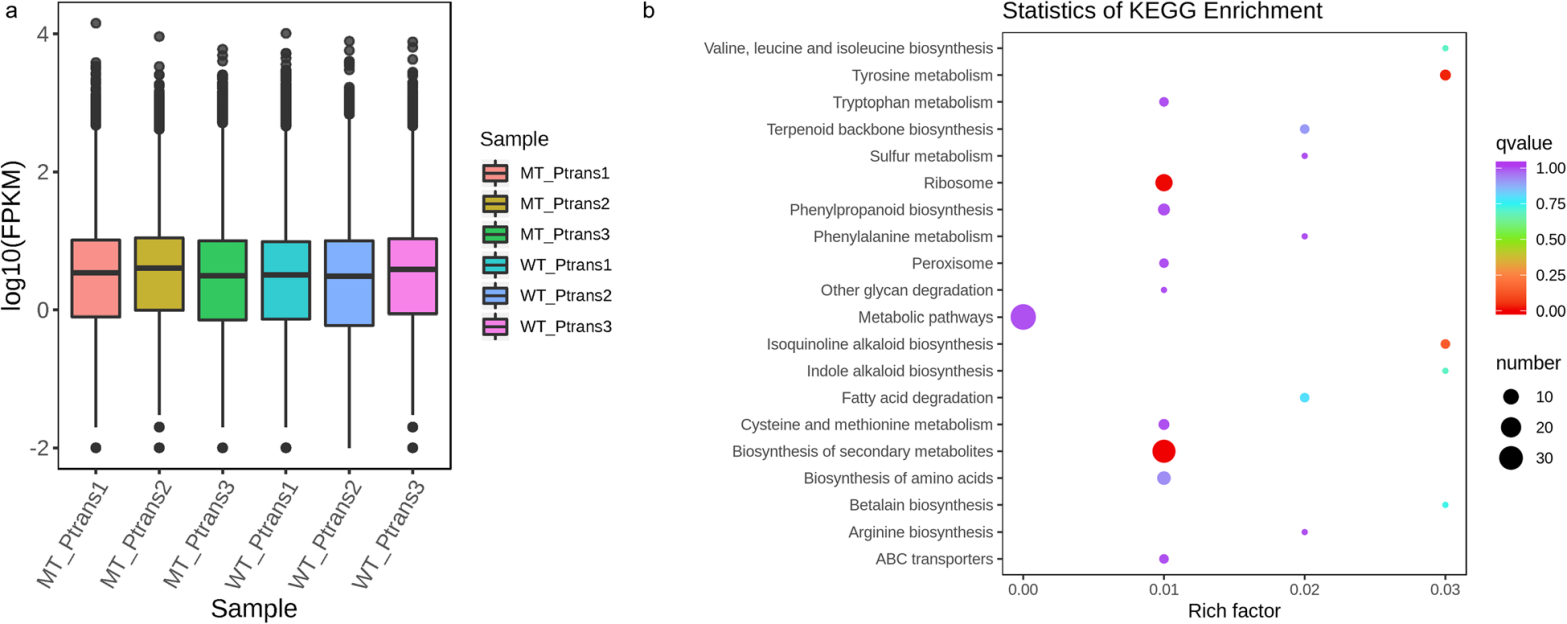
**a** Discrete and overall degree of gene expression in the replicates of both WT and MT Aifen No. 1 banana pseudostems. **b** Scatter plot of 20 most enriched KEGG pathways between WT and MT Aifen No. 1 banana pseudostems. The degree of enrichment is shown by Rich factor, Q-value, and the number of genes enriched in each pathway

We investigated the changes in gene expression profiles in the pseudostems of WT and MT plants. With three biological replicates, the transcriptome sequencing of the six samples yielded a total of 56.78 Gb clean data with an average of 94.46% bases scoring Q30 (Additional Table 1). Of the total clean reads, 83.68 to 90.25% were unique matches with Musa genome *(Musa acuminata* DH Pahang V2 and *Musa balbisiana* DH PKW) [18, 27]. Overall, the Fragments Per Kilobase of Transcript per Million Fragments Mapped (FPKM) for MT was higher as compared to WT, when FPKM > 1 was used as a threshold to determine the gene expression (Fig. 2a). Pearson correlation coefficient (PCC) between WT and MT replicates ranged from 0.25 to 0.87 (Additional Fig. 1). This is expected since MT was obtained from WT by tissue culture and probably, only a few genes were mutated. We also observed that there is a high intra-varietal diversity for pseudostem transcriptome. Therefore, we would recommend more replicates in future studies related to pseudostem growth.

### Differentially expressed genes between WT and MT

DESeq2 analysis revealed that a total of 127 genes were differentially expressed (DEG) between WT and MT; 25 and 102 DEGs showed decreased and increased expression in MT as compared to WT, respectively. These results imply that very few genes are involved in pseudostem height in banana. The gene expression cluster analysis based heatmap of each differential group is presented in Additional Fig. 2. Of the DEGs, only five genes were exclusively expressed in WT. The NR annotation showed that these genes were predicted as BIG GRAIN 1-like E isoform X1 (*gene-C4D60_Mb04t03190*), protein SRG1-Like (*gene-C4D60_Mb11t04180*), S-norcoclaurine synthase 1-like (*gene-C4D60_Mb11t04200*), an uncharacterized protein (*Ma02_g04590*), and a stellacyanin isoform X1 (*Ma05_g16810*) (Additional Table 3). Only one gene (*gene-C4D60_Mb07t24780*) was exclusively expressed in MT banana with a log2fold value of 7.082. This gene was predicted as an integral part of the membrane. The Kyoto Encyclopedia of genes and Genomes (KEGG) analysis revealed that the top-five key biological pathways that were significantly enriched in WT vs MT banana were metabolic pathways, biosynthesis of secondary metabolites, ribosomes, tyrosine metabolism, and phenylpropanoid biosynthesis (Fig. 2b; Additional Fig. 3).

### Differentially expressed genes related to plant hormones

Regulation of GAs causes banana plants to exhibit the dwarf phenotype [1]. Hence, it is important to check if the expression of GA biosynthesis pathway-related genes were differentially modulated between WT and MT. We found that an N-acetylglucosaminyl-phosphatidylinositol biosynthetic protein gpi1-like (gibberellin-regulated protein 6) (*Ma06_g04390*) was highly up-regulated in MT as compared to WT. We also observed a probable 2-oxoglutarate-dependent dioxygenase (*Ma07_g15430*) which was up-regulated in MT. Owing to cross-talk between BR and GAs, we looked for expression of related genes in our DEGs and observed the up-regulation of two BRI genes (gene-*C4D60_Mb10t18040* and *Ma10_g11870*). Because LOB domain-containing genes are targeted by DELLAs, the decrease in the expression of one LOB domain-containing gene was differentially expressed [28]. DELLAs interact with TFs such as TCP and Jasmonate ZIM domain-containing proteins (JAZ). In this regard, we found that two genes were up-regulated in MT; one JAZ (*gene-C4D60_Mb07t13750*) and one TCP (*Ma03_g32580*). Among the highly expressed genes in MT, the increase in the expression of two interleukin-1 receptor-associated kinase 4 genes (*gene-C4D60_Mb08t19920* and *gene-C4D60_Mb10t14420*) with fold change values of 172.317 and 93.8, respectively, is an important observation. Previously it is known that plant stature-related receptor-like kinase 2 (PSRK2) acts as a factor that determines stem elongation toward gibberellins response in rice [29].

Mevalonate pathway has a minor contribution to the GA biosynthesis pathway [10]. In this regard, we observed that two GHMP kinase genes (*Ma08_g34050* and *gene-C4D60_Mb08t33270*) having mevalonate kinase activity (based on GO annotation) had increased expression in MT. It is also known that cytosolic acetyl-CoA is further involved in the biosynthesis of mevalonate-derived isoprenoids [30]. One gene (*gene-C4D60_Mb01t19300*) annotated as cytosolic acetyl-CoA showed decreased expression in MT.

Four members of the ethylene responsive factor (ERF) (ERF/AP2) family (*Ma02_g02820, Ma05_g04880, Ma09_g22060,* and *gene-C4D60_Mb02t03000*) showed increased expression in MT as compared to WT. The GCN5-related N-terminal acetyltransferases (GNATs) are directly involved in the transcriptional regulation of meristem regulatory genes such as WUSCHEL [31]. We observed an increased expression of one GNAT (*gene-C4D60_Mb10t18260*) in our transcriptome dataset (Additional Table 3).

In terms of the role of auxin in BR synthesis and signaling, as well as hypocotyl elongation, the expression of two genes (*gene-C4D60_Mb10t21290* and *Ma10_g08300*) was relatively increased in MT suggesting a role of auxin in banana pseudostem elongation. One of the exclusively expressed genes in WT was BIG GRAIN 1-like, which is known to be involved in auxin transport [32]. The expression of a HB-HD-ZIP TF (*gene-C4D60_Mb09t13640*) was increased in MT. Our transcriptome data showed that two genes (*Ma06_g03830* and *gene-C4D60_Mb08t08250*) belonging to ABC transporter G and C families, respectively, were up-regulated in MT suggesting the involvement of polar auxin transport in stem elongation [33].

Among the highly expressed genes in MT, the second highest expression was noted for a TIFY-5A-like protein (*Ma07_g01220*). This gene is known for its role as a repressor of Jasmonate responses [34, 35]. Three genes annotated as phospholipase A1-II 5-like showed increased expression in MT (*gene-C4D60_Mb07t09300*, *Ma02_g10300*, *and Ma08_g26980*). Phospholipase As are involved in root elongation as well as in auxin signaling [36].

### Differentially expressed genes related to Cell Wall, cell growth, and stem height

A dedicated mechanism involving pectin methylesterase inhibitors (PMEI) controls the cell-wall rigidity and decreases its visco-elasticity [37]. The lower expression of one PMEI (*gene-C4D60_Mb03t05630*) in MT might be considered a strategy towards stem elongation [38]. We also observed reduced expression of cellulose synthase-like protein (*Ma08_g05160*) and a member of glycosyltransferase like family 2 (cellulose synthase-like protein E2), while two probable mannan synthases (gene-*C4D60_Mb07t06270* and *Ma07_g22600*) showed increased expression in MT (Additional Table 3).

We observed that the expression of a fatty-acyl-CoA synthase was increased in MT banana. Furthermore, the expression of four caffeic acid 3-O-methyltransferases (COMT) (*Ma09_g18140*, *Ma09_g18200*, *gene-C4D60_Mb09t20170*, and *gene-C4D60_Mb09t20150*) was increased in MT [39, 40].

We also observed the relatively lower expression of genes related to starch degradation i.e. inactive beta-amylase 9 (*Ma03_g08740*) [41], 1-acyl-sn-glycerol-3-phosphate acyltransferase PLS1 which has also been reported to be involved in growth of the cucumber seedlings [42], and a stellacyanin, 3-ketoacyl-CoA synthase 4-like (*Ma05_g16810*) (key enzyme catalyzing the first reaction in fatty acid elongation and determining the substrate specificity) [43] (Additional Table 3). Apart from hormones and cell-wall structural control of cell elongation, it is known that codeine 3-O-demethylases are linked to plant cell division and stem elongation via the demethylation of mono-, di-, and tri-methylated lysine-4 residues of histone subunit 3. We found that a codeine 3-O-demethylase (*gene-C4D60_Mb11t04180*) had almost no expression in MT [44] (Additional Table 3). GABA accumulation is known to cause cell elongation defects. Our transcriptome data showed exactly this feature where one transmembrane amino acid transporter protein (*gene-C4D60_Mb01t32380*; probable GABA transporter 1) had lower expression in MT (Additional Table 3) [45]. White-brown complex homolog protein 15 (*Ma06_g03830*) (ABC transporter) showed increased expression in MT. This gene is highly expressed in developing cotton fiber cells and is correlated with cotton fiber elongation [46].

A probable carboxylesterase 18 was highly expressed in MT as compared to WT (92.9-fold increase in the expression). These genes have been functionally associated with plant height in maize through genome-wide association studies [47]. The 9-cis-epoxycarotenoid dioxygenase (*Ma04_g38030*) was highly expressed in MT. These are implicated in early seedling growth after germination and have shown significantly longer roots and shoots as compared to non-transformed plants [48].

We also found three aminocyclopropanecarboxylate oxidases (ACO) (*gene-C4D60_Mb05t08270*, *Ma05_g09360*, and *gene-C4D60_Mb06t13490*) that showed increased expression in MT as compared to WT. ACO1 was shown to affect internode elongation at the heading stage in rice [49].

It is known that ribosomal subunit proteins regulate leaf morphology as well as plant architecture [50]. In this regard, we observed that a ribosomal protein S7p/S5e (*gene-C4D60_Mb00t01000*) had lower expression MT with a log2fold change value of − 5.149 (Additional Table 3).

### Validation of DEGs by quantitative RT-PCR

**Fig. 3:**
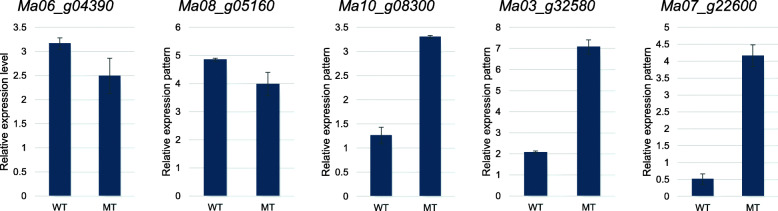
qRT-PCR analysis of the five selected differentially expressed genes in WT and WT banana pseudostems

In order to confirm the validity of the RNA-Seq based transcript abundance of genes, a qRT-PCR analysis of five DEGs was performed; *Ma10_g08300* (membrane transport proteins), *Ma03_g32580* (TCP family transcription factor), *Ma07_g22600* (probable mannan synthase 11 isoform X1), *Ma06_g04390* (gibberellin-regulated protein 6), and *Ma08_g05160* (cellulose synthase-like protein). The *Actin* and *Histone* genes were used as internal controls. The qRT-PCR profiles of the five selected genes matched with the RNA-seq based results, which validates the useability of the Illumina sequencing results (Fig. 3).

### Differentially accumulated metabolites between WT and MT

**Fig. 4:**
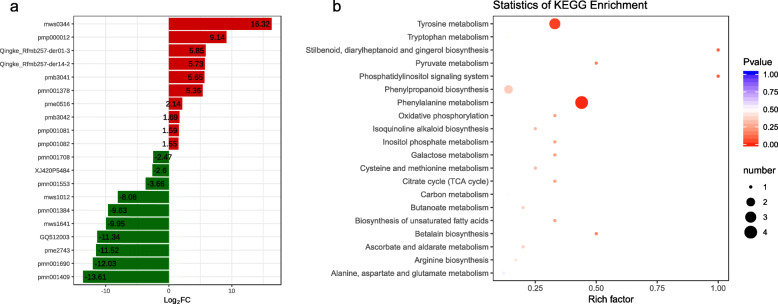
**a** Top-10 up-accumulated (shown in red bars) and top-10 down-accumulated metabolites (shown in green bars) in WT vs MT Aifen No.1 banana pseudostem. **b** Scatter plot of 20 KEGG pathways between WT and MT Aifen No. 1 banana pseudostems to which the differentially accumulated metabolites were enriched. The degree of enrichment is shown by Rich factor, *P*-value, and the number of metabolites enriched in each pathway

We also studied the WT and MT banana pseudostem metabolome to understand the role of metabolites in stem elongation. In total, we detected 412 metabolites. These detected metabolites could be grouped into 10 major classes (Additional Table 4). For the evaluation of differences in metabolite ion intensity between WT and MT, we applied the PLS-DA model. The established PLS-DA model showed good fitness (Additional Fig. 4). Forty-eight metabolites were differentially accumulated in WT vs MT Aifen no. 1 banana pseudostem (Additional Fig. 5; Additional Table 5). Seventeen metabolites belonging to six classes i.e. amino acid and its derivatives (1), flavonoids (5), lignans and coumarins (4), organic acids (1), phenolic acids (3), and others (3), were higher in MT. The down-accumulated metabolites belonged to class alkaloids, amino acid and its derivatives, flavonoids, lignans and coumarins, lipids, nucleotide and its derivatives, organic acids, phenolic acids, tannins, terpenes, and others. The hierarchical clustering clearly differentiated the metabolites (Additional Fig. 6). The top-10 down-accumulated metabolites in MT were Asiatic acid (pmn001708), hydroxyanigorufone (XJ420P5484), cimidahurinine (pmn001553), coumarin (mws1012), isochlorogenic acid C (pmn001384), lumazine (mws1641), fer-agmatine (GQ512003), N-phenylacetylglycine (pme2743), 3-hydroxy-4-isopropylbenzylalcohol 3-glucoside (pmn001690), and plantainoside A (pmn001409). The top-10 up-regulated metabolites in MT were D-Xylonic acid lithium salt (mws0344), scutellarin (pmp000012), pinoresinol-hex (Qingke_Rfmb257-der01–3), pinoresinol-aceGlu (Qingke_Rfmb257-der14–2), tricin O-saccharic acid (pmb3041), terpineol monoglucoside (pmn001378), inositol (pme0516), tricin 5-O-hexoside (pmb3042), sudachiin B (pmp001081), and sudachiin C (pmp001082) (Fig. 4a). We further functionally annotated the differentially accumulated metabolites (DAMs) in KEGG database and found that the most significantly enriched pathways were phenylalanine metabolism, phenylpropanoid biosynthesis, and tyrosine metabolism (Fig. 4b).

### Combined Transcriptome and Metabolome analyses

**Fig. 5:**
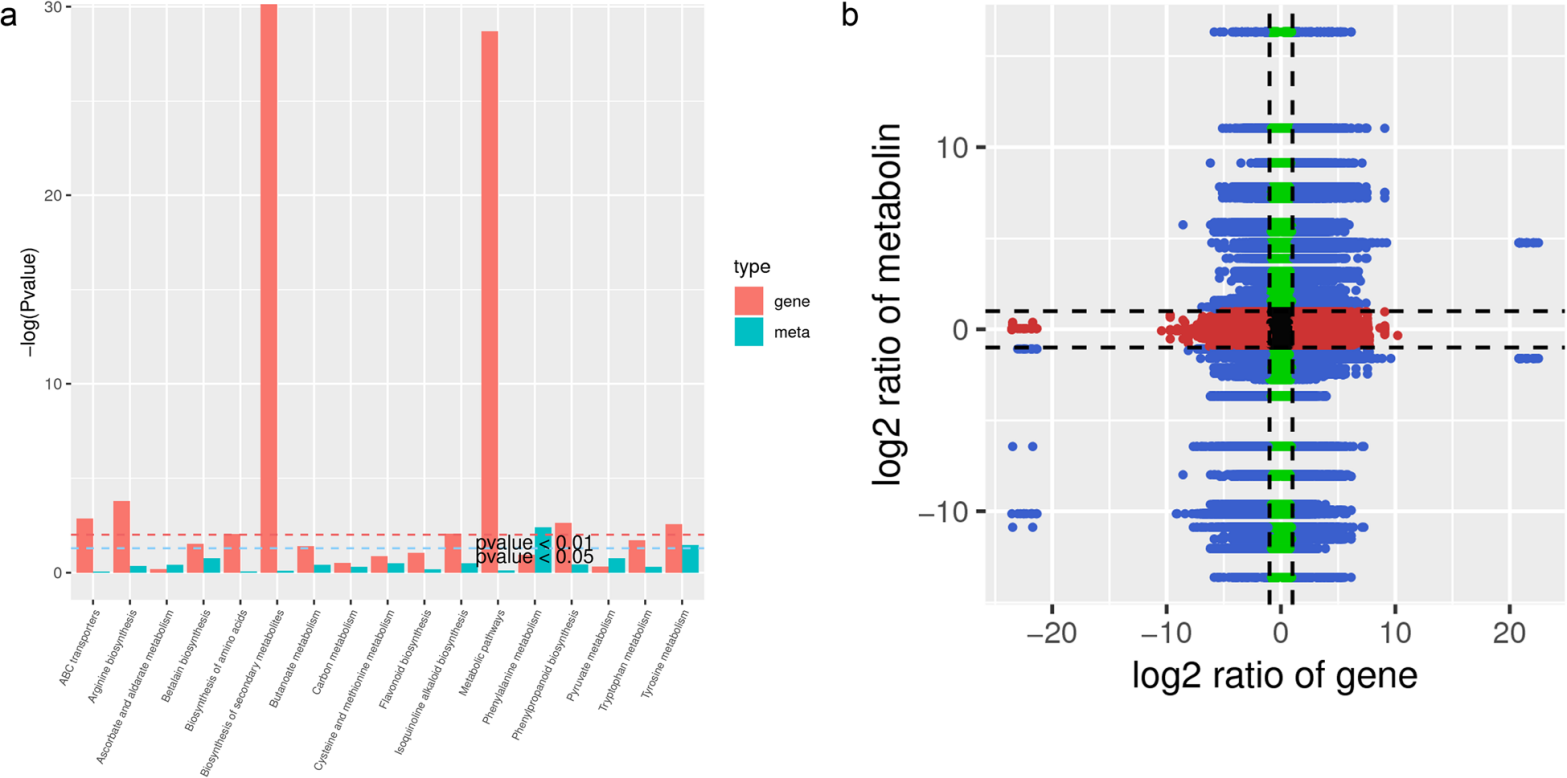
Joint analysis of DEGs and DAMs between WT and MT Aifen No. 1 banana pseudostems. **a** Joint KEGG enrichment *p*-value histogram, **b** Pearson correlation coefficient of DEGs and DAMs in WT vs MT banana pseudostem represented as a nine-quadrant diagram. Black dots = unchanged genes/metabolites, green dots = DAMs with unchanged genes, red dots = DEGs with unchanged metabolites, blue dots = DEGs and DAMs. The Pearson’s correlation coefficient is > 0.8 throughout the nine-quadrant graph

To establish a relationship between different levels of molecules i.e. transcriptome and metabolome, we first visually compared the separate results of PCA. The PC1 of DEGs and DAMs had 23.07 and 26.11% variation, respectively. The PC2 of DEGs and DAMs showed 36.41 and 37.51% variation, respectively, suggesting that the amount of variation was more or less same (Additional Fig. 7). The co-joint KEGG pathway analysis showed that the DEGs and DAMs were mapped onto fifteen pathways (Additional Table 6; Fig. 5a). The co-joint KEGG pathway enrichment analysis showed that the same pathways were enriched as of transcriptome or metabolome based individual enrichment analysis. The significantly enriched pathways are shown in Fig. 5a. Log2 conversion data for the DAMs and DEGs were selected that had a PCC > 0.8. We then generated nine-quadrant diagrams to visualize corresponding variations between DAMs and DEGs having PCC as given above (Fig. 5b; Additional Fig. 8). Transcript-metabolite correlation was determined by constructing subnetworks. These networks revealed both the regulatory and synthetic characteristics of genes and metabolites (Additional Fig. 9). The correlation networks showed that transcripts and metabolites were correlated in four pathways i.e., Ascorbate and aldarate metabolism, cysteine and methionine metabolism, phenylpropanoid biosynthesis, and biosynthesis of amino acids. Furthermore, the relatively higher number of DAMs and DEGs in the 3rd and 7th quadrant indicates that the differential expression pattern of genes and differential accumulation of metabolites are consistent; genes and metabolites have a positive correlation, and the changes in metabolites may be positively regulated by genes (Fig. 5a).

## Discussion

Since the early banana improvement breeding efforts through tissue culture, dwarfism and stem height variants have been commonly observed [5]. Remarkable advances have been made to understand the mechanisms governing plant height in banana as well as other fruit trees. However, stem height/elongation is a complex physiological and biological process that involves various biological pathways (GA metabolism, BR, ABA, and auxin), mechanical properties of cell-walls, and coordinated signaling between the growing cells/tissues [3]. Here, we studied the DEGs and DAMs based on combined transcriptome and metabolome profiling of WT and MT of Pisang Awak Aifen No.1 for insights into the mechanisms related to banana plant height. We discuss the main pathways to which the studied DEGs and DAMs were associated.

It is known through loss-of-function mutations in the key GA biosynthesis regulatory enzymes display dwarf phenotypes [51]. Apart from biosynthesis-related genes, DELLAs are the key genes in the GA signal transduction pathway and DELLA deletion mutations in maize and brassica have been associated with dwarfism [52, 53]. The differential expression of DELLAs was not recorded in the comparative transcriptome analysis between WT and MT banana pseudostems. Similar observations have been previously recorded in autotetraploid apple where authors did not find differential regulation of DELLAs. However, the authors concluded that the stem height was being regulated by GA [54]. We also propose similar GA regulated pseudostem height regulation in MT. This proposition is based on many observations. First, the 36.66 fold up-regulation of one gibberellin regulated protein (*Ma06_g04390*) is an important observation, as in cucumber hypocotyl, this gene has been reported to encode a classical arabinogalactan protein and is involved in stem elongation [55]. Second, we also observed an increased expression of two other genes that are induced by GA i.e. interleukin-1 receptor-associated kinases with very high fold change values. This observation is in agreement with the fact that in rice, a receptor-like kinase named PSRK2 was induced by GA and was only found in the stem [56]. Third, the observation that a probable 2-oxoglutarate-dependent dioxygenase (*Ma07_g15430*) showed increased expression, further supports our proposition of possible involvement of GA in pseudostem height. This gene belongs to 2OG-Fe (II) oxygenase superfamily and its homologs are known for their role in catalyzation of latter reactions in the GA biosynthesis pathway [9]. Fourth, it is known that brassinosteroids (BRs) and GA are two predominant hormones regulating plant cell elongation. A defect in either of these leads to reduced plant growth and dwarfism. BRI is the major receptor of BR and overexpression of BRI1 in Arabidopsis has shown increased cell elongation and increased number of brassinolide binding sites in membrane fractions [57]. The significant increase in the expression of two BRI1 genes in MT suggests that BR is increasing pseudostem height by cell elongation. This observation is consistent with the results that decreased BR content in the autotetraploid apple caused dwarf phenotype [54]. Furthermore, BR catabolism is activated by LOB domain-containing proteins which are particularly important in the control of stem elongation [58]. We observed that one LOB domain-containing gene had higher expression in WT. LOB domain-containing genes are targeted by DELLA proteins. LOB genes are categorized as organ boundary genes. Thus the organ boundary genes could regulate stem growth by restricting signals that control stem elongation, such as BR, which further regulates GA synthesis [28]. It has been already established in rice that BR modulates GA biosynthesis and hence regulates cell elongation. Fifth, the regulation of JAZ and TCP further supports the possible involvement of GA in pseudostem height regulation. Finally, the mevalonate pathway has also contribution to the GA biosynthesis pathway because geranylgeranyl diphosphate is synthesized by either a mevalonate-dependent or a non-mevalonate pathway [10]. The increased expression of a gene associated with mevalonate kinase activity suggests that GA metabolism is influencing the phenotype and the GA biosynthesis is possibly due to changes in the mevalonate-dependent pathway [9].

GAs also work in coordination with BR, ABA, auxin, and other hormones for stem growth regulation [59, 60]. Auxin biosynthesis, transport, and sensitivity are associated with cell elongation in plants [61, 62]. Auxin has a specific role in epidermis during hypocotyl elongation is and controls BR synthesis and signaling [63]. Epidermis coordinates auxin-induced stem growth hence changes in auxin flux in cells could be linked with cell elongation via auxin efflux carrier proteins (also known as PINs) [14, 16]. The up-regulation of two PINs in MT suggests that a similar mechanism might be present in banana stem. This observation is supported with the fact that we noticed up-regulation of one HD-zip protein in MT. Previously; links have also been established between auxin and the members of HD-zip proteins. The HD-zip gene (*AtHB2*) controls the elongation of hypocotyl depending on the auxin transport system [64, 65]. Furthermore, ABC transporters also play roles in auxin transport. The dwarf phenotype in Arabidopsis has also been implicated to ABC transporters owing to their involvement in auxin transport [33]. Therefore, any up-regulation of ABC transporter in MT will further verify that auxin influx in elongated pseudostem could be a possible regulatory mechanism. In this study two the ABC transporter had higher expression. In addition, ABC transporter was one of the significantly enriched pathways in transcriptome as well as metabolome (Fig. 2b). Additionally, the relatively higher expression of phospholipases and a patatin-like protein 2 is quite relevant to these results because it has been established that phospholipases (including patatin-related phospholipases) have functions in auxin signal transduction [66]. Together, these findings suggest that auxin flux in the pseudostem was significantly affected and could lead to elongation of the stem.

Phospholipase-As have not been well characterized regarding cell elongation in Arabidopsis [67]. Therefore, the higher expression of phospholipases also suggests possible role in cell elongation in MT. Stem elongation or plant height is accomplished by cell expansion/elongation, which is a tightly regulated process. In Arabidopsis, cell elongation is further responsible for hypocotyl growth, while in cotton it is attributed to fiber elongation. The exclusive accumulation of scutellarin (*pmp000012*) in MT suggested that MT pseudostems are experiencing elongation. Scutellarin is a parahormone that promotes root elongation [68] (Additional Table 5). Overall, all plant organs owe their final size to a period of significant cell elongation [69]. Regarding cell elongation, our results showed that a probable GABA transporter had a lower expression, while a white-brown complex homolog protein 15 showed increased expression. These observations indicate that like Arabidopsis and cotton, banana cell elongation is also regulated in a complex manner [45, 46]. As the size of a plant cell is determined by the extent of the surface of its wall, cell elongation may be defined also as any permanent increase in the surface of the cell wall [70]. Therefore, cell wall modification is an important consideration when comparing WT and MT banana pseudostems. We further checked whether cell-wall elasticity or porosity is affected during pseudostem elongation in banana. Previous investigations in Arabidopsis showed that cellulose composition and cell-wall thickness were affected very little during stem elongation. The role of pectin and cellulose is quite established in control of internode elongation [38]. Our observations in MT pseudostem suggest that banana might adopt a strategy to control the cell-wall rigidity and possibly reduce its visco-elasticity by down-regulating PMEI. The increased expression of probable mannan synthases and reduced expression of cellulose-synthase-like protein might be associated with protein-mediated changes in cell-wall extensibility driven by the circadian clock which should be further validated by future studies (Additional Table 3) [71, 72]. Furthermore, the increased expression of four COMTs is consistent with the previous reports on high lignin content, mechanical strength, and stem strength in lodging resistant wheat [39, 40]. In this regard, the 81.968 fold higher accumulation of D-xylonic acid was an important observation with no accumulation in WT (Additional Table 5). D-xylonic acid has been found as a predominant constituent of the hemicellulose in pomegranate thus suggesting that large-scale cell wall modification is underway in MT [73]. This is further evident from the 57 and 53 fold up-accumulation of major lignans i.e. pinoresinol-hex and pinoresinol-aceGlu, respectively, in MT (Additional Table 5) [74].

**Fig. 6:**
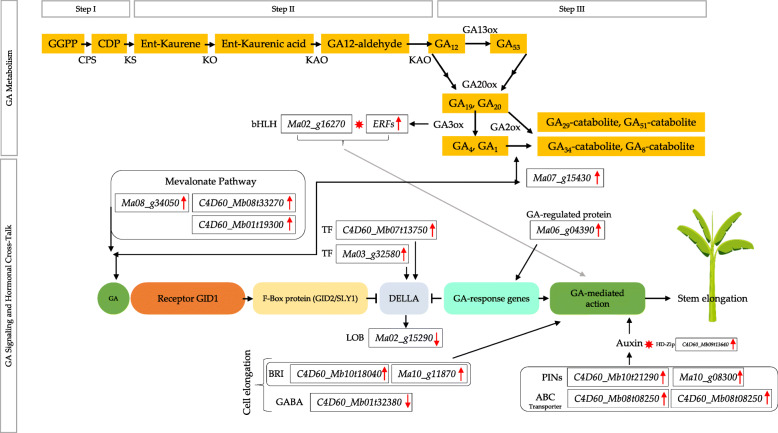
Proposed model of the hormonal cross talk leading to the increase in pseudostem height in MT Aifen No. 1 banana. Red arrow indicates the up (↑) or down (↓) regulated genes and red stars indicate that a putative interaction exists

Based on our results and known roles of different hormones and their cross talk with TFs, we propose that interaction of BRs, auxins, and ERFs with GA metabolism and signaling is likely to be responsible for the increase in pseudostem height of MT Aifen No.1 banana (Fig. 6). This novel understanding opens new research avenues for the further in-depth investigation of the separate and interactive role of each specified hormone and associated proteins or TFs.

## Conclusions

In this study, we aimed at delineating possible mechanisms governing pseudostem height in banana by employing a combined transcriptome and metabolome analysis of a mutant and its wild type plant. At the metabolic level, phenylpropanoid biosynthesis, tyrosine metabolism, and phenylalanine metabolism pathways showed differential regulation. At the transcriptome level, we observed that biosynthesis of secondary metabolites, metabolic pathways, ribosomes and tyrosine metabolism were significantly enriched. The higher expression of a gibberellin regulated protein, interleukin-1 receptor-associated kinases, a probable 2-oxoglutatrate-dependent dioxygenase, and BRI genes in MT coupled with the changes in the expression of genes associated with mevalonate pathway indicates that GA is an important contributor in pseudostem height regulation. Our results also confirmed the known coordinated role of GAs with BR, ABA, auxin, and other hormones for stem growth regulation. The pseudostem height in MT was also being regulated by the changes in the expression of cell-wall related genes, cell elongation, and expansion.

## Methods

### Plant material

The plant material was a wild type (WT) of a dwarf banana ‘Aifen No. 1’ (*Musa* spp. Pisang Awak sub-group ABB) and its semi-dwarf mutant (MT). The MT was obtained from tissue-culture induced somaclonal variations in Aifen No. 1 WT at Institute of Fruit Tree Research, Guangdong Academy of Agricultural Sciences, Guangzhou, China by the corresponding author of this article (Professor Ou Sheng). The specimens used in this study have neither been reported nor submitted in any herbarium. The MT and WT were grown in nutrient-soil for four weeks of rooting and two days of hardening. After this, the seedlings were grown to reach five to six leaf stage (6 weeks old). The relative humidity, temperature, and photoperiod were 28 °C, 60 ~ 80%, and 12 h (1500 ± 200 lx), respectively. After six weeks, the banana plantlets had five to six true leaves. Triplicate pseudostems for WT as well as for MT were then cut and used for transcriptome and metabolome analyses (Fig. 1a).

### RNA extraction, library preparation, and Illumina Hiseq sequencing

We used the standard procedures for the extraction of total RNAs from WT and MT pseudostems, which involved the use of Spin Column Plant total RNA Purification Kit (Tiandz, Beijing, China) [75]. Further, the RNAs’ purity was assessed, the extracted RNAs were quantified, and their integrity was checked. Following these steps, sequencing libraries were prepared and sequenced on the Illumina HiSeq platform (Illumina Inc., San Diego, USA) as reported earlier [76, 77].

### Sequencing data analysis

We employed FastQC (http://www.bioinformatics.babraham.ac.uk/projects/fastqc/) to process the sequencing reads and obtain clean reads followed by determining the GC content distribution. HISAT2 was used to perform sequence alignment between clean reads and the reference genomes i.e. Musa genome *(Musa acuminata* DH Pahang V2 and *Musa balbisiana* DH PKW) [18, 27, 78, 79]. The comparison efficiency (percentage of mapped reads to clean reads) was calculated followed by the calculation of the position distribution statistics of the reads on different chromosomes [80]. The regional distribution of the reads was compared and plotted as a chart. The results were visualized in IGV software [81]. The quality of the transcriptome library was assessed by randomization test of mRNA fragmentation followed by an insertion length check by calculating the distance between the start and end points of the reads on the reference genome at the ends of the insert. Gene expression was quantified as Fragments Per Kilobase of transcript per Million fragments mapped (FPKM). The gene expression data was then used to express the trend of gene abundance and overall distribution of gene expression, calculate correlation, and principal component analysis (PCA). For functional annotation, we aligned the genes with various databases such as KEGG [82], GO [83], Clusters of Orthologous Groups of proteins (COG) [84], and EuKaryotic Orthologous Groups (KOG) [85] using BLAST [86] with a threshold of E-value < 1.0 × 10^− 5^.

### Identification of differentially expressed genes, pathway enrichment, and real-time qRT-PCR

The differential gene expression analysis and enrichment analyses were performed as previously described [76]. Briefly we used DESeq2 to differential expression genes (DEGs) after normalizing the read counts with the filtering criteria of fold change and false discovery rate (FDR) or > 2 and *p* < 0.01, respectively [87]. Hierarchical cluster analysis was done and represented as heat maps using Corset (https://code.google.com/p/corset-project/).

The identified DEGs were mapped on pathways in KEGG in KOBAS2.0 [88]. Care was taken to minimize the false positive prediction for which we employed FDR correction with *p* < 0.05.

After analyzing the expression of genes, we selected five DEGs (Additional Table 7) that showed varied expression in WT and MT pseudostems and performed quantitative real-time PCR (qRT-PCR) on a Rotor-Gene 6000 machine (Qiagen, Shanghai, China) as following. cDNAs were synthesized from RNA using the High Capacity cDNA Reverse Transcription Kit (Applied Biosystem, MA, US). Two constitutively expressed genes i.e. *Actin* (F:CTCCTATGTTGCTCGCTTATG, R:GGCTACTACTTCGGTTCTTTC) and Histone *H3.3* (F:GCGGTAATAGGAGTGAAGTC, R: TCAGCCTCAGCCAATGGCAC) were used as reference [89–91]. The temperature profiles, reagents, and volumes for PCR reactions were similar to previously described conditions [76, 92].

### Metabolome analysis

Six weeks old pseudostems of the tissue cultured MT and WT Aifen No. 1 banana that were stored at − 80 °C were processed at Wuhan MetWare Biotechnology Co., Ltd. (www.metware.cn). The remaining steps in processing the samples, extraction, and detection of the metabolites were done as reported earlier [93].

### Metabolomics data analysis

The intensities of metabolites were submitted and processed in Analyst 1.6.1 software (AB SCIEX, Ontario, Canada) and metabolites with missing values were considered to be lower than the detection limit and replaced with a minimum recorded value. The ion intensities were normalized by log transformation, the metabolite abundance was calculated by using Dunnett’s test, and multiple testing was controlled by FDR. To obtain the maximum differences between the treatments, we used the partial least squares-discriminant analysis (PLS-DA) and variable importance in projection > 1 to screen DAMs was used to calculate the relative importance of the identified DAMs to the PLS-DA model. Other analyses including PCA, Hierarchical Clustering, and pathway enrichment were completed by using R (www.r-project.org) as reported earlier [20, 24, 82, 94].

### Combined Transcriptome and Metabolome analyses

In addition to individual analyses for transcriptome sequencing and metabolome profiling, we performed co-joint analyses on DEGs and DAMs to determine the degree of enrichment of pathways. The Corson program in R was used to calculate the PCC of genes and metabolites and a correlation coefficient cluster heatmap of DEGs and DAMs was drawn. Gene-metabolite networks with a PCC > 0.8 were used to construct the transcript-metabolite network.

## Supplementary Information

**Additional file 1: Figure 1**. Pearson correlation between WT vs MT banana pseudostem replicates. **Figure 2**. Heatmap hierarchical clustering of differential expressed genes in WT vs MT banana pseudostems where abscissa indicates the sample names (WT and MT), and the ordinate indicates the differential expressed genes. **Figure 3**. KEGG enrichment analysis of differentially expressed genes in WT vs MT banana pseudostems. **Figure 4**. Partial least squares-discriminant analysis. **Figure 5**. Variable importance in projection score plot. **Figure 6**. Heatmap hierarchical clustering of differentially accumulated metabolites. **Figure 7**. Principal Component Analysis (A) differentially expressed genes and (B) differentially accumulated metabolites. **Figure 8**. Correlation coefficient cluster heat map of differentially expressed genes and differentially accumulated metabolites having Pearson’s correlation coefficient > 0.8. **Figure 9**. Transcript-metabolite correlation network representing DAMs and DEGs involved in MT banana stem elongation. The KEGG pathways are given on the top of each network. The gene and metabolite IDs correspond to Additional Table 3 and Additional Table 5, respectively.

**Additional file 2: Table 1**: Summary of sequencing output statistics. **Table 2**: Mapping results of RNA-Seq data from WT and MT banana pseudostem. **Table 3**: Complete list of differentially expressed genes in WT vs MT banana pseudostem. **Table 4**: List of all detected metabolites in WT vs MT pseudostem. **Table 5**: List of differentially expressed metabolites in WT vs MT banana pseudostem. **Table 6**: Co-joint KEGG analysis. The list shows the pathways onto which the differentially regulated genes and differentially accumulated metabolites were mapped. **Table 7**: Primer sequences of the genes used for qRT-PCR.

## Data Availability

RNA-seq data is available at the SRA database in National Center of Biotechnology Information with the accession number PRJNA609266 (https://www.ncbi.nlm.nih.gov/sra/?term=PRJNA609266).

## Abbreviations

GA: Gibbrellic acid
BR: Brassinosteroids
ABA: Abscisic acid
SAM: Shoot apical meristem
RZ: Rib zone
TF: Transcription factor
TCP: Class 1 teosinte branched 1
LOB: Lateral organ boundaries
WT: Wild type
MT: Mutant
FPKM: Fragments Per Kilobase of Transcript per Million Fragments Mapped
DEG: Differentially expressed
KEGG: Kyoto Encyclopedia of genes and Genomes
JAZ: Jasmonate ZIM domain-containing proteins
PSRK2: plant stature related receptor-like kinanse 2
GO: Gene Ontology
ERF: Ethylene responsive factor
GNAT: GCN5-related N-terminal acetyltransferases
PMEI: Pectin methylesterase inhibitors
COMT: Caffeic acid 3-O-methyltransferases
PLS-DA: Partial least squares-discriminant analysis
PCA: Principle component analysis
DAM: Differentially accumulated metabolites
PCC: Pearson correlation coefficients
COG: Clusters of Orthologous Groups of proteins
KOG: EuKaryotic Orthologous Groups
qRT-PCR: Quantitative Real-time PCR
