# Supplementary material for: Transcriptome and metabolome profiling provide insights into molecular mechanism of pseudostem elongation in banana
Source: BMC Plant Biol. 2021 Mar 1;21:125. doi: 10.1186/s12870-021-02899-6 (PMC7923470; doi:10.1186/s12870-021-02899-6)

**Additional figure 1** Pearson correlation between the expression in WT vs MT banana pseudostem replicates. The red colour in the scale represent maximum correlation (1) and reducing red intensity represents lower correlation.

**
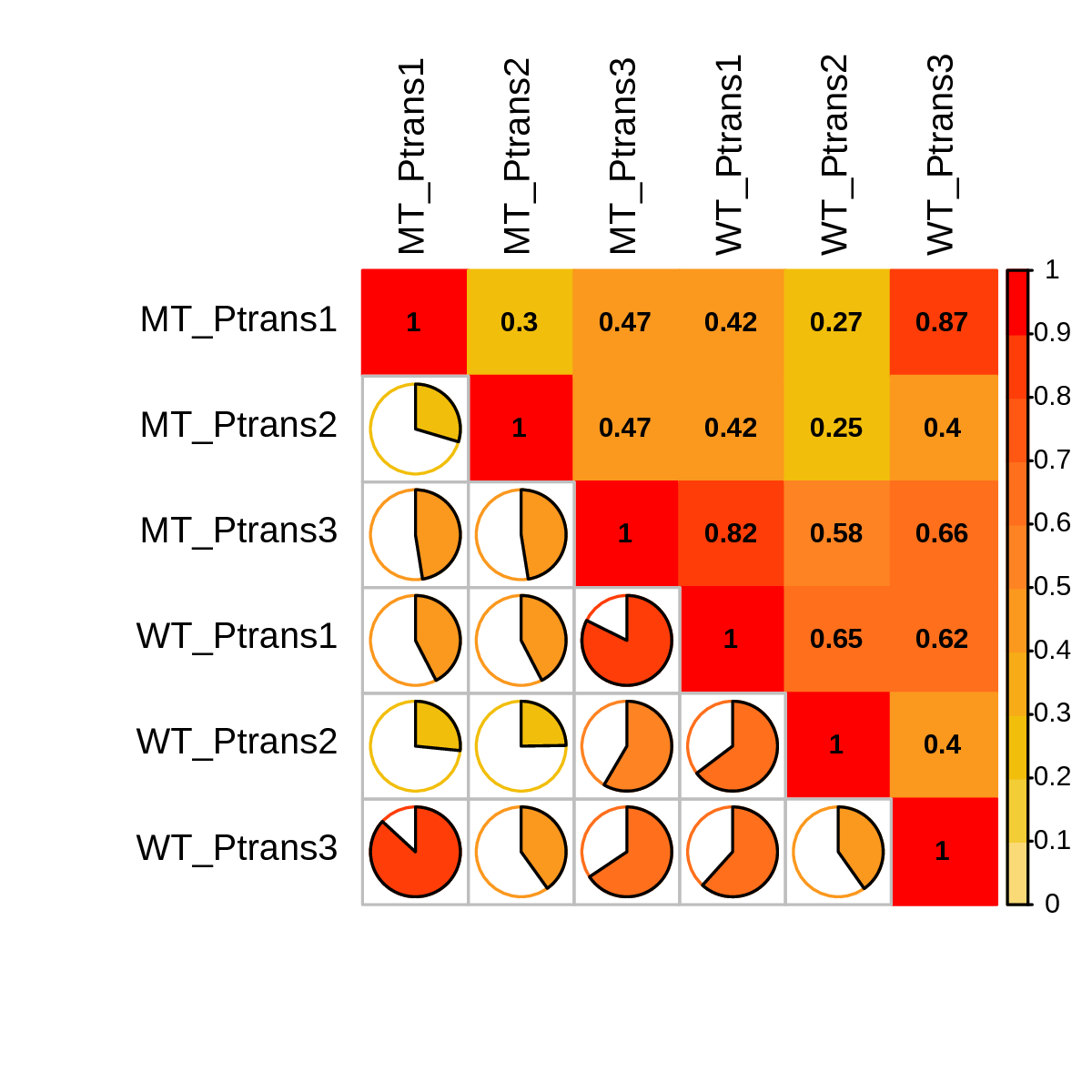
**

**Additional figure 2** Heatmap hierarchical clustering of differential expressed genes in WT vs MT banana pseudostems where abscissa indicates the sample names (WT and MT), and the ordinate indicates the differential expressed genes. Green and dark orange bars on the top represent MT and WT, respectively. The dark brown and dark green in the scale on the right represent maximum and minimum expression of genes in each replicate of WT and MT.

**
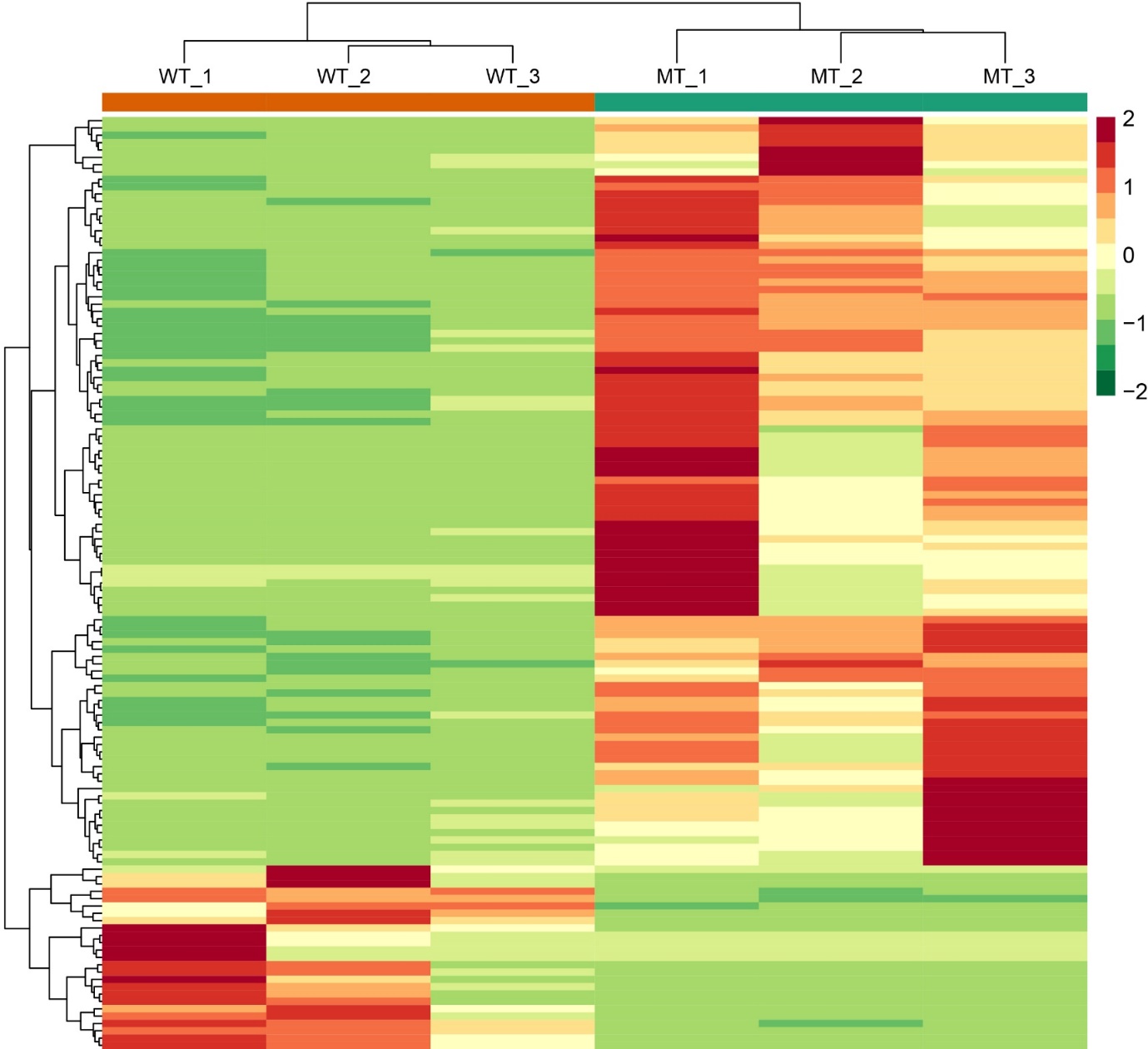
**

**Additional figure 3** KEGG enrichment analysis of differentially expressed genes in WT vs MT banana pseudostems. Green bar represent pathway related to genetic information processing and the light purple colour represents the metabolism related pathways.
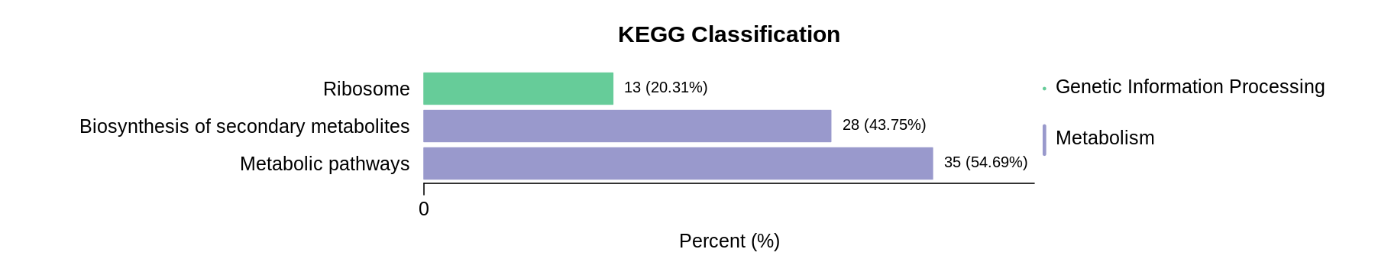


**Additional figure 4** Partial least squares-discriminant analysis of metabolites. In the abscissa, p1 represents the predicted principal component, o1 represents the orthogonal principal component, and the ordinate represents the corresponding R2X, R2Y and Q2.


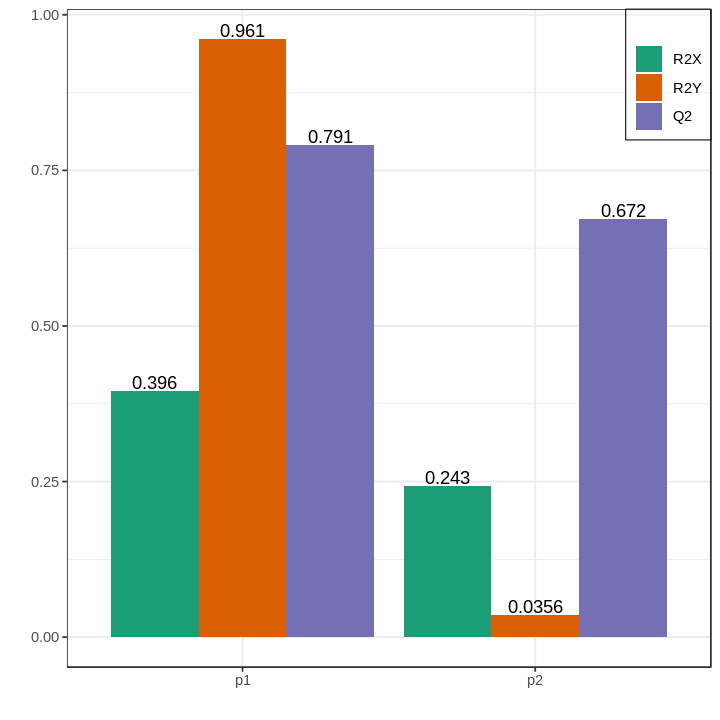


**Additional figure 5** Variable importance in projection score plot of differentially accumulated metabolites. Each point in the volcano map represents a metabolite, the abscissa represents the logarithmic value of the quantitative difference multiple of a certain metabolite in the two samples; the ordinate represents the VIP value. The greater the absolute value of the abscissa, the greater the multiple difference in the accumulation level between the two samples; the greater the ordinate value, the more significant the differential accumulation, and the more reliable the differentially accumulated metabolites screened. The green dots in the figure represent down-regulated differentially accumulated metabolites, the red dots represent up-regulated differentially accumulated metabolites, and the gray represents detected but not significantly different metabolites.

**
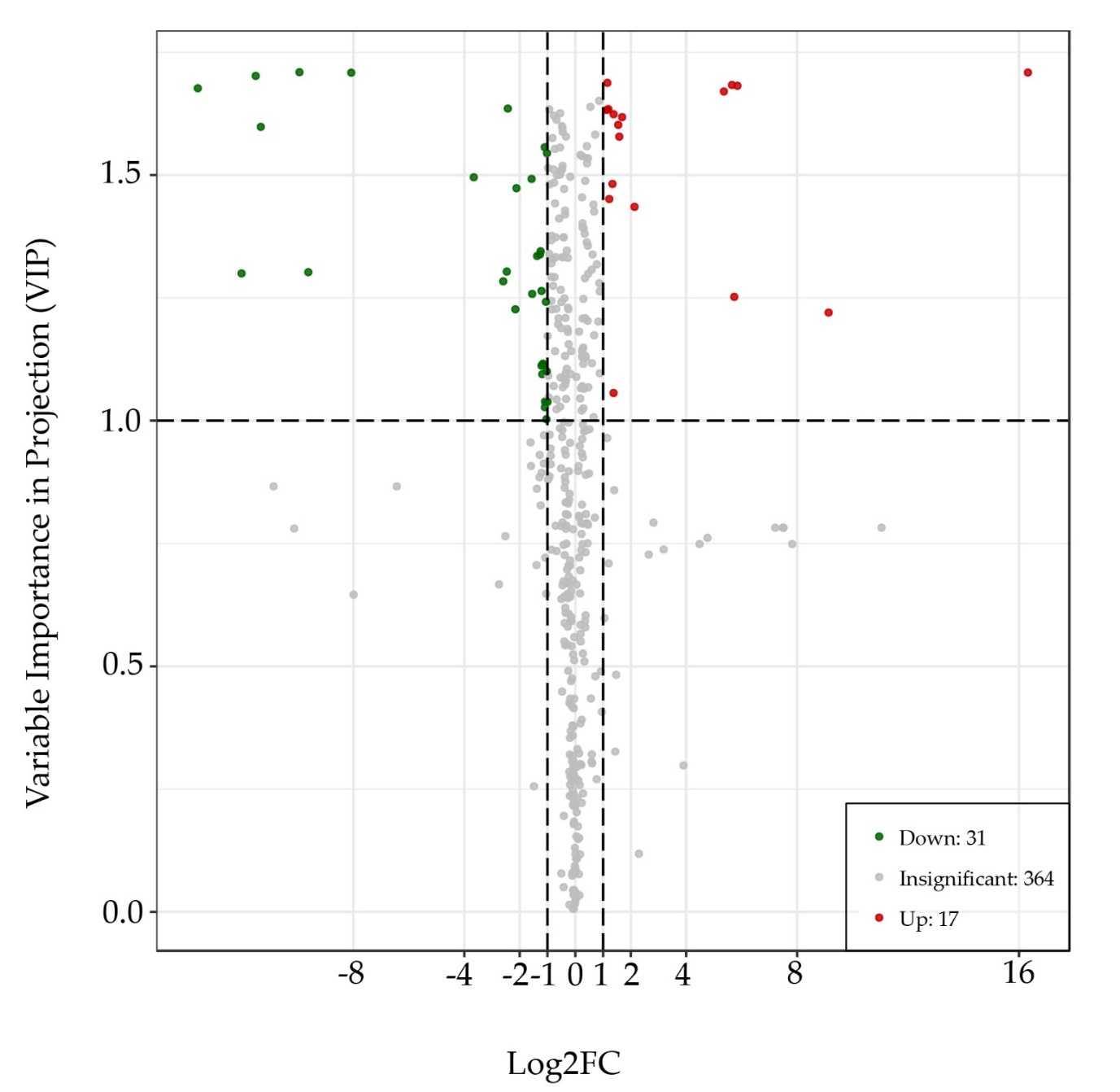
**

**Additional figure 6** Heatmap hierarchical clustering of differentially accumulated metabolites. The dark brown and dark green in the scale on the right represent maximum and minimum concentration of metabolites in each replicate of WT and MT.

**
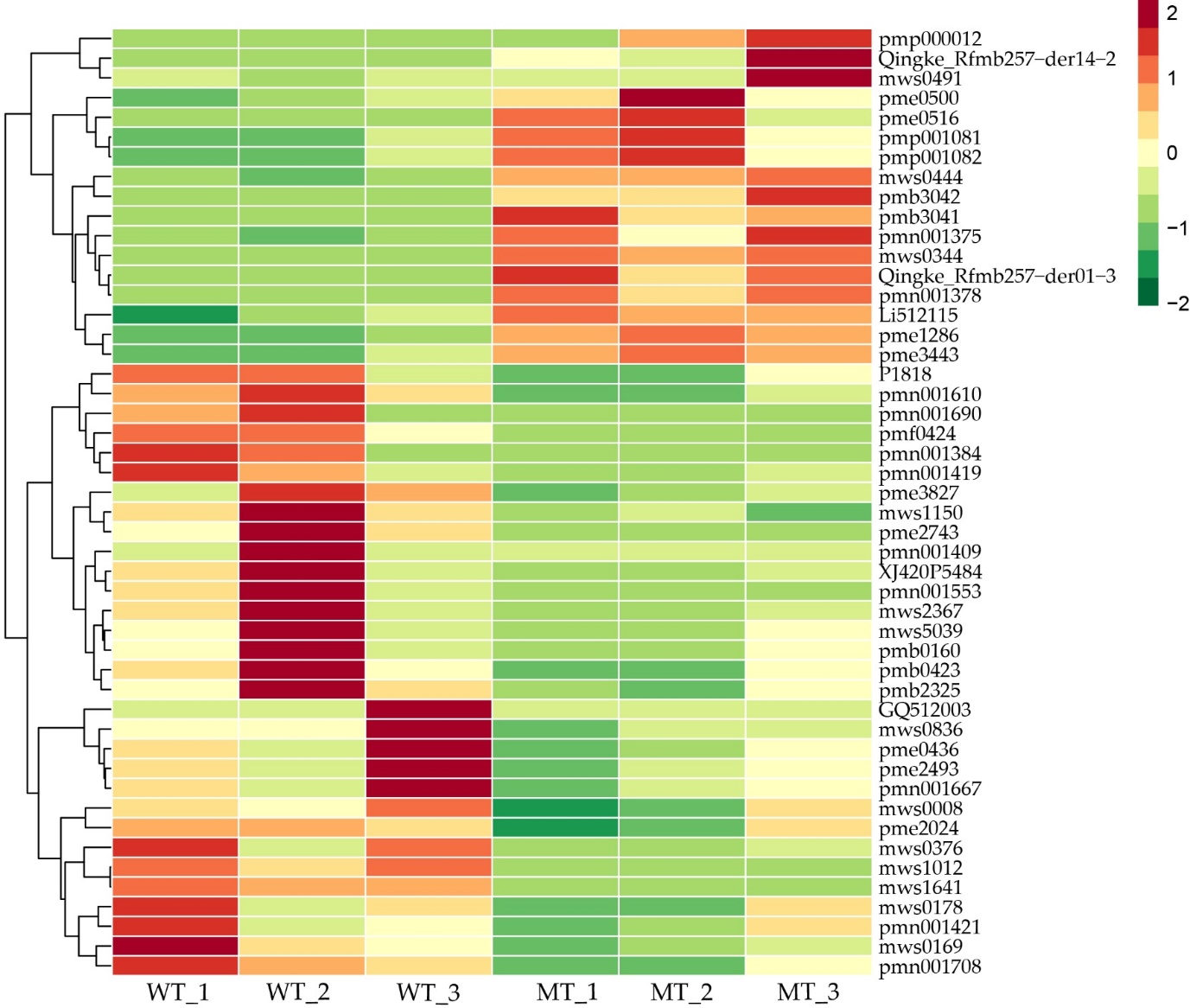
**

**Additional figure 7** Principal Component Analysis **a** differentially expressed genes and **b** differentially accumulated metabolites. The colors in the plot correspond to the colour groups given on the right side of the plot.

1. Principal Component Analysis of DEGs


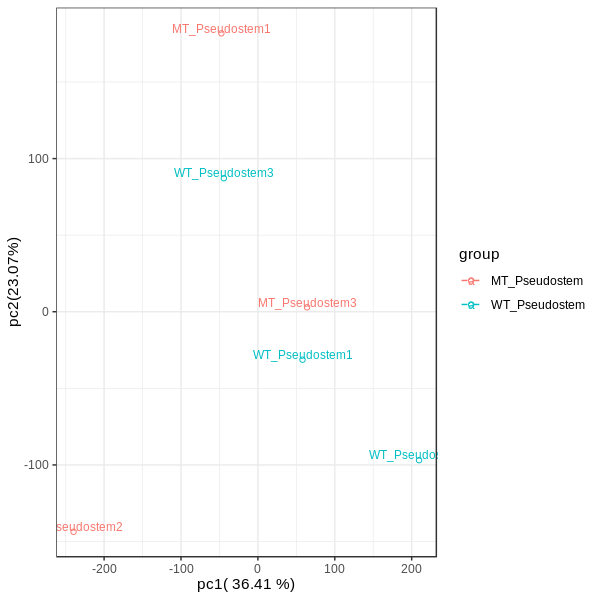


1. Principal Component Analysis of DAMs


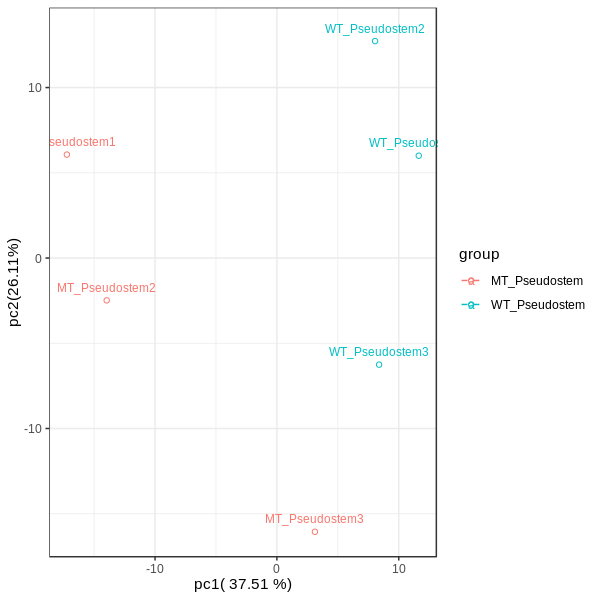


**Additional figure 8** Correlation coefficient cluster heat map of differentially expressed genes and differentially accumulated metabolites having Pearson’s correlation coefficient >0.8. The colors in top of the heatmap represent different groups of metabolites. The colour description of metabolite group is given as a key on the right side of the figure. The heatmap scale colour intensity shows that highest expression is dark brown and lowest is dark green.

**
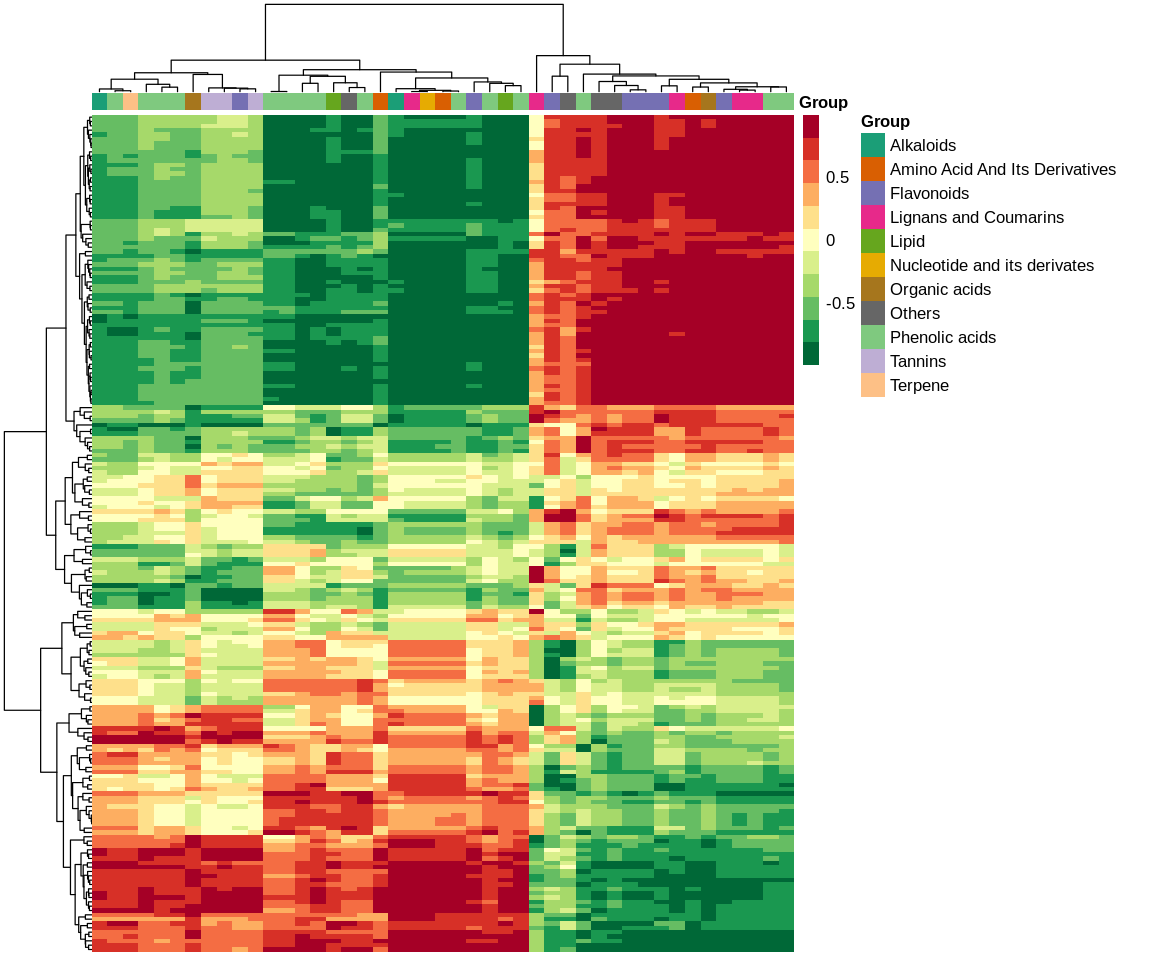
**

**Additional figure 9** Transcript-metabolite correlation network representing DAMs and DEGs involved in MT banana stem elongation. The KEGG pathways are given on the top of each network. The gene and metabolite IDs correspond to Table S3 and Table S5, respectively.


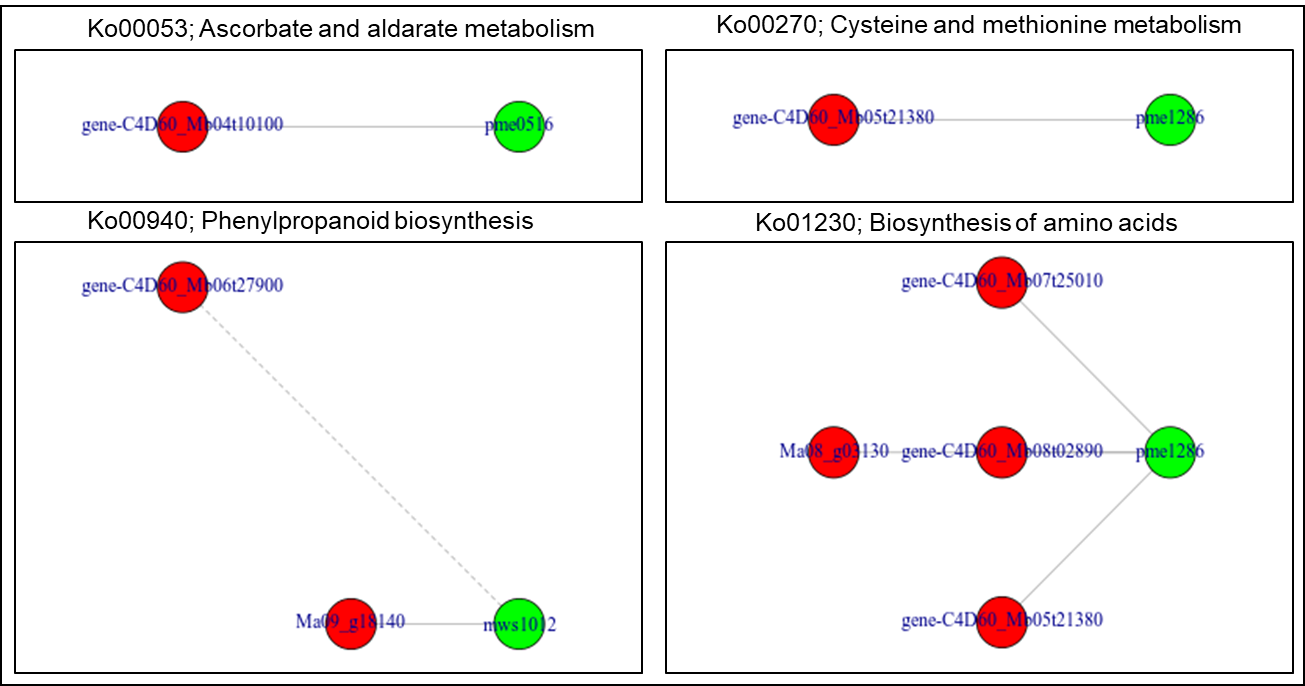

Supplement: Supplementary file 1 — Additional file 1: Figure 1. Pearson correlation between WT vs MT banana pseudostem replicates. Figure 2. Heatmap hierarchical clustering of differential expressed genes in WT vs MT banana pseudostems where abscissa indicates the sample names (WT and MT), and the ordinate indicates the differential expressed genes. Figure 3. KEGG enrichment analysis of differentially expressed genes in WT vs MT banana pseudostems. Figure 4. Partial least squares-discriminant analysis. Figure 5. Variable importance in projection score plot. Figure 6. Heatmap hierarchical clustering of differentially accumulated metabolites. Figure 7. Principal Component Analysis (A) differentially expressed genes and (B) differentially accumulated metabolites. Figure 8. Correlation coefficient cluster heat map of differentially expressed genes and differentially accumulated metabolites having Pearson’s correlation coefficient > 0.8. Figure 9. Transcript-metabolite correlation network representing DAMs and DEGs involved in MT banana stem elongation. The KEGG pathways are given on the top of each network. The gene and metabolite IDs correspond to Additional Table 3 and Additional Table 5, respectively. [file 12870_2021_2899_MOESM1_ESM.docx]
